# Comparison of green and synthetic silver nanoparticles in zein‐based edible films: Shelf‐life study of cold‐stored turkey breasts

**DOI:** 10.1002/fsn3.3661

**Published:** 2023-09-10

**Authors:** Ammar Mohammed Ali Eesa, Behnaz Bazargani‐Gilani, Shaimaa Obaid Hasson

**Affiliations:** ^1^ Department of Food Hygiene and Quality Control, Faculty of Veterinary Science Bu‐Ali Sina University Hamedan Iran; ^2^ Department of Medical Biotechnology, College of Biotechnology Al‐Qasim Green University Babylon Iraq

**Keywords:** chemical methods, green tea leaf extract, synthetic silver nanoparticles, turkey breast meat, zein edible film

## Abstract

This study aimed to compare the effect of zein edible film containing silver nanoparticles produced by green tea leaf extract (Z‐gAgNPs) with zein film containing synthetic silver nanoparticles (Z‐AgNPs) on the shelf life of turkey breast during refrigerated storage. The produced silver nanoparticles were analyzed using dynamic light scattering (DLS), UV–Vis spectroscopy, X‐ray diffraction (XRD), scanning electron microscope (SEM), and atomic force microscope (AFM). According to the obtained results, the green fabricated silver nanoparticles (gAgNPs) showed higher polydispersity index (PDI), stability, homogeneity, spherical shape without cavity, and lower size compared to the synthetic silver nanoparticles (AgNPs). The studied treatments were divided into four groups, including 1‐ control (C) (turkey breast meat without packaging), 2‐ Z (turkey breast meat packaged with zein film), 3‐ Z‐AgNPs (turkey breast meat packaged with zein film containing 0.5% (w/w) of AgNPs), and 4‐Z‐gAgNPs (turkey breast meat packaged with zein film containing 0.5% (w/w) of gAgNPs). The treatments were analyzed for 12 days with 3‐day intervals in refrigerator conditions. In general, the measurement of total viable count, total volatile basic nitrogen (TVB‐N), and pH values showed that Z‐gAgNPs film significantly (*p* ≤ .05) delayed the spoilage of the studied samples until the end of the 12th day of storage and Z‐AgNPs, Z, and C treatments were in the next ranks, respectively. It is concluded that the biofabricated silver nanoparticles using green tea leaf extract have more appropriate physicochemical features and higher efficiency compared to the synthesized silver nanoparticles using chemical methods in zein edible films in improving the shelf life of the cold‐stored turkey breast meat and can be introduced as a promising alternative to the plastic packaging.

## Graphical abstract

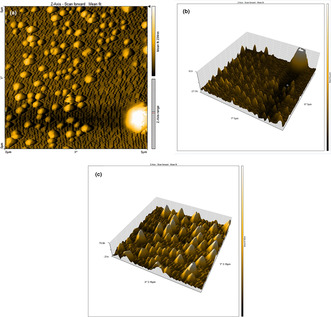

## 1 INTRODUCTION

Turkey is a proper choice among the other kinds of meat products for consumers due to its low fat, cholesterol, and high content of protein. Turkey meat is rich in minerals, such as calcium, phosphorous, potassium, and B group vitamins, including B1, B2, B6, and B12 (Milani et al., 2020; Sayadi et al., 2021). But, the high perishability of meat products has limited their use. High moisture, pH, and protein of turkey meat have created an ideal environment for microbial growth and spoilage reactions, such as oxidation and nutrient breakups (Keykhosravy et al., 2020). Consumers' demands for natural food products using natural and biodegradable packaging have increased. Biopolymers are the main basis for the production of the natural packaging. Among different biopolymers, Zein is known as an ideal substance given its properties, such as excellent plasticity, biodegradability, and harmlessness (Zolfaghari et al., 2023). Zein is a hydrophobic protein in corn endosperm and a byproduct of corn milling. Zein is a part of corn prolamin, which is commercially produced from corn gluten during the starch production process (Barkhordari & Bazargani‐Gilani, 2021; Yıldırım‐Yalçın et al., 2021). Zein films have a good barrier against gases, but it has a high water vapor permeability and is brittle (Zhan et al., 2020). Corn zein is rich in alanine, leucine, proline, and glutamic acid (Cui et al., 2020). Strengthening the edible films with antimicrobial agents is an effective approach to improving its physicochemical and preservative features (Zhan et al., 2020). Nanoparticles are effective antimicrobial agents that have promising applications in edible films and coatings. Silver nanoparticles (AgNPs) are among the most widely used nanoparticles in active packaging (Shankar et al., 2021; Zhan et al., 2020). Different chemical and physical methods have been used for the synthesis of nanoparticles (Vishwanath & Negi, 2021). However, the physical methods have low efficiency; as a result, they are not economical, and using chemical techniques leads to the production of toxic byproducts (such as sodium nitrate, diborane (B_2_H_6_), and sodium borohydride in synthesis of the silver nanoparticles) that can seriously threaten the environment (Hassanisaadi et al., 2022; Vishwanath & Negi, 2021). Therefore, using the cost‐effective, eco‐friendly, efficient, and safe methods is essential for the synthesis of nanoparticles. Recently, using green techniques in the fabrication of nanoparticles by plant extracts, fungus, and bacteria has been widely considered (Jardón‐Romero et al., 2022; Kumar et al., 2021). Green tea (*Camellia sinensis*) leaf extract is the new approach for the biofabrication of AgNPs. There are a lot of studies about the biosynthesis of silver nanoparticles using green tea extract (GTE). High content of reductant ingredients in green tea extract can reduce silver nitrate to silver nanoparticles (Ali et al., 2022; Göl et al., 2020). Since there is no study “to the author's knowledge” to compare the biochemical properties of synthetic and green fabricated silver nanoparticles, we intended to compare the efficiency of green AgNPs with synthetic ones in zein edible film in the shelf‐life study of cold‐stored turkey breast in this study.

## 2 MATERIALS AND METHODS

### 2.1 Materials

Zein, sodium carbonate, magnesium oxide, boric acid, ethanol, sulfuric acid, and hydrochloric acid were purchased from Sigma Aldrich Chemical Company (Steinheim, Germany). Tween 80, sodium bromide, peptone water, and plate count agar culture mediums were obtained from Merck Company (Darmstadt, Germany).

### 2.2 Preparation of GTE

The dried green tea leaves of Lahijan were purchased from local market (Hamedan, Iran) and ground by a blender. Then, the powder was mixed with deionized water at a ratio of 1:10 (w/v). The obtained solution was boiled for 10 min and cooled at room temperature. Next, the solution was filtered with Whatman No. 1 filter paper (Ali et al., 2022). The resulting aqueous extract was stored in a refrigerator (4 ± 1°C) for next use.

### 2.3 Green fabrication of silver nanoparticles

A total volume of 750 mL of silver nitrate (10 mM) was added (drop by drop) using automatic burette (HSCG‐1655 GelsonLab) into the 25 mL of the green tea extract on the magnetic stirrer (PIT300, Poly Ideal Tajhiz, Tehran, Iran) at 30–50°C for 120 min in 800 rpm. After discoloring the solution to brown, the produced gAgNPs were concentrated, purified, and rinsed with deionized water. Then, they were dried and analyzed by the relevant tests (Ali et al., 2022).

### 2.4 Chemical synthesis of silver nanoparticles

0.002 mL of sodium borohydride was mixed with 30 mL of the distilled water. Next, 1 mL of silver nitrate was added (drop by drop) to the obtained solution until yellow color appeared in the solution. Then, the resulting solution was added (drop by drop) to 20 mL of trisodium citrate on the stirrer (PIT300, Poly Ideal Tajhiz, Tehran, Iran) in 150 rpm. The final solution was centrifuged at 25,200 *g* for 15 min. Then, the obtained AgNP sediment was rinsed with distilled water three times and was used for the experiment (Vahedikia et al., 2019).

### 2.5 Characterization of AgNPs and gAgNPs

#### 2.5.1 UV–visible spectroscopy

In this method, the absorbance spectra of the synthesized silver nanoparticles are recorded in the range 400–500 nm. The UV–visible spectra of AgNPs and gAgNPs were obtained using UV–visible spectrophotometer (Shimadzu‐UV1700, Japan) in the range 200–800 nm. Blank is the used GTE without silver nanoparticles (Hassanisaadi et al., 2022).

#### 2.5.2 Dynamic light scattering (DLS)

The size (diameter), polydispersity index (PDI) (homogeneity), and zeta potential (stability) of the synthesized AgNPs and gAgNPs were measured by the dynamic light scattering (DLS) technique. The produced silver nanoparticles were mixed with distilled water with a concentration of 1 mg/mL and analyzed using DLS method (Zetasizer Nano‐ZS, Germany) (Kumar et al., 2021).

#### 2.5.3 X‐ray diffraction (XRD)

XRD technique was used for identifying the amorphous or crystalline nature of the samples. The samples were analyzed by X‐ray diffractometer (Rigaku Ultima IV, Japan) in the 2*θ* angle and the range 10–80° scanning rate of 0.5°/s (Ali et al., 2022).

#### 2.5.4 Scanning electron microscope analysis (SEM)

The SEM images of the gAgNPs were provided using a scanning electron microscope (MIRA 3 TESCAN) in the voltage of 15 kV and magnifications of 10, 20, 35, 70, and 135 k × in the scales of 5, 2, 1 μm, 500, and 200 nm, respectively (Singh et al., 2018; Widatalla et al., 2022).

#### 2.5.5 Atomic force microscope analysis (AFM)

In three‐dimensional images of AFM, morphology features (topography and roughness) and exact size of the samples are determined. The AFM images of the AgNPs were provided by the atomic force microscope (C‐2M‐FP, ARA, Iran) (Ghanbarzadeh & Oromiehi, 2008; Singh et al., 2018).

### 2.6 Film preparation

Corn zein powder (10% w/v) was heated in 96% ethanol for 1 h at 80°C. Glycerol 20% and Tween 80 (2%) were used as the stabilizer and emulsifier in the films. In the case of films containing gAgNPs and AgNPs, 0.5% of each was added to the related solutions. The films were produced by the casting method so that the required amount of the prepared solutions was poured into the sterile Petri plates; then, the mold was gently shaken to distribute the solution evenly. Finally, to desiccate the film, the molds were kept on a flat surface at room temperature for 24 h. Upon complete drying, the films were slowly removed from the mold (Zolfaghari et al., 2023).

### 2.7 Preparation of the treatments

The fresh turkey breast meat was purchased from the market, Hamedan, Iran, and was immediately transported to the laboratory beside the ice pack. Then, in completely sterile conditions under a biological containment hood (Fan Azma Gostar, Tehran, Iran), the turkey meat was cut into 10 g pieces and each one was separately put inside the films; after thermal stitching of the films by sealer machine (PFS‐200, Yatong plastic), each of the samples was placed separately in sterile zip packs at a temperature of 4°C and microbial and chemical properties were evaluated on days 0, 3, 6, 9, and 12 of the storage periods. The studied treatments included: 1‐ control group (turkey meat without packaging), 2‐ Z group (turkey meat packaged with Z film alone), 3‐ Z‐AgNPs group (turkey meat packaged with Z film containing 0.5% AgNPs), and 4‐ Z‐gAgNPs group (turkey meat packaged with Z film containing 0.5% gAgNPs).

### 2.8 Microbial analysis

#### 2.8.1 Sample preparation

Using a scalpel blade and sterile forceps, 10 g of the meat was separated and placed in a sterile zip pack containing 90 mL of 0.1% peptone water solution. It was well homogenized by the stomacher device (Model 400, Circulator Lab Blender, US) for 60 s. Then, using 0.1% peptone water solution, serial dilutions were prepared.

#### 2.8.2 Total mesophilic bacteria

Total mesophilic bacteria counting was performed by a surface cultivation method in PCA (plate count agar) culture medium. In this method, 0.1 mL of the desired dilution was cultured on the plates. Then, the plates were placed in an incubator at 37°C for 48 h. After this period, the colonies on the plates were counted and the number of bacteria was reported as log 10 CFU/g (Yousef et al., 2022).

### 2.9 Chemical analysis of the Turkey breast meat

#### 2.9.1 pH measurement

For pH measurement, 5 g of the samples along with 25 mL of distilled water was homogenized for 60 s. Then, the pH of the samples was measured using a pH meter (Denver, Germany) at room temperature (Barkhordari & Bazargani‐Gilani, 2021).

#### 2.9.2 Total volatile basic nitrogen (TVB‐N)

To measure the total volatile basic nitrogen, 10 g of the homogenized meat was mixed with 300 mL of the distilled water and 2 g of the magnesium oxide, then poured into a Kjeldal flask and heated. The vapors obtained from the distillation were trapped into a beaker containing 25 mL of 3% boric acid and a few drops of reagent. Distillation continued until the volume of boric acid and reagent became 50 mL and the color of the solution changed to green. In the end, the flask content was titrated with 0.5 M of sulfuric acid. The amount of TVBN was calculated based on the amount of sulfuric acid. TVB‐N values were calculated in mg of nitrogen/ 100 g of the sample (Tavakkoli et al., 2020).

### 2.10 Statistical analysis

All tests were performed in three replications based on a completely randomized design. SPSS version 26 (IBM SPSS statistics V. 26) was used for the statistical analysis of data. Statistical comparison was done by one‐way ANOVA at 95% confidence level and Tukey's test was used to mean comparison. The graphs were drawn by Microsoft Excel 2016 software (Microsoft, Redmond, WA, USA) and all results were presented as mean ± standard deviation.

## 3 RESULTS AND DISCUSSION

### 3.1 UV–visible spectroscopy

**FIGURE 1:**
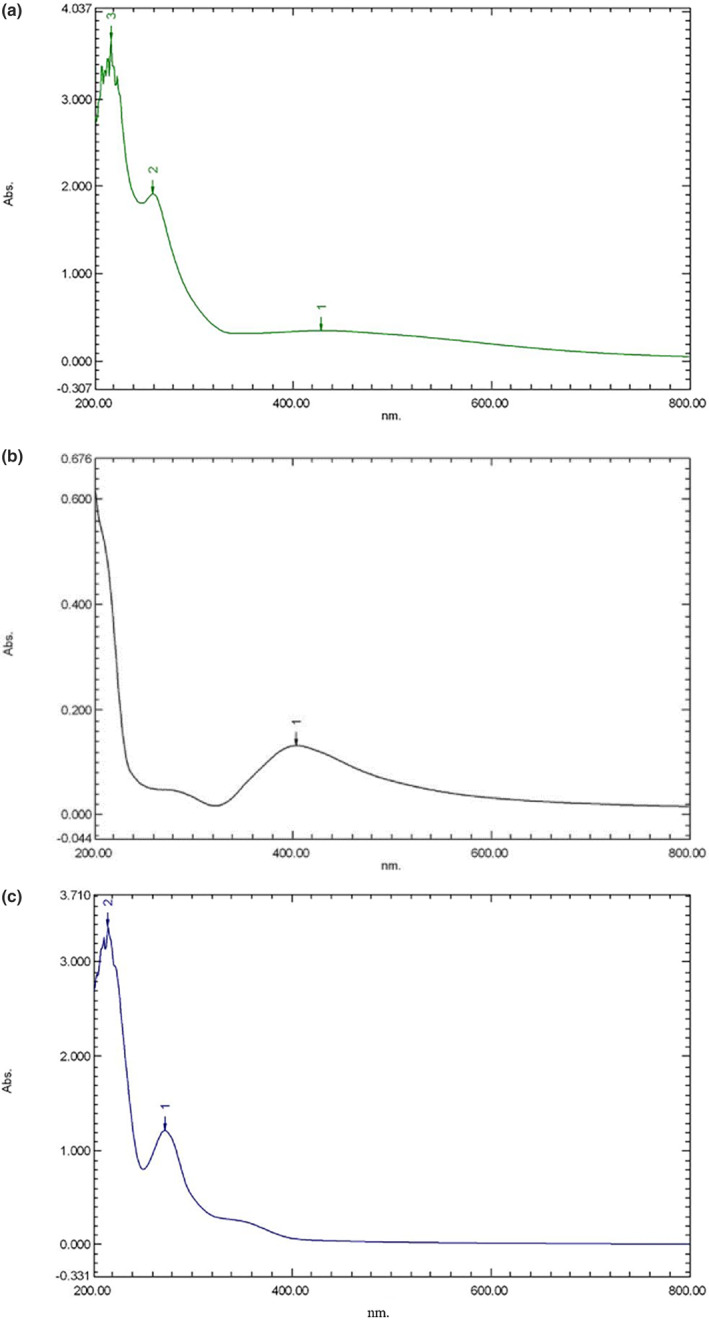
(a–c) UV‐visible spectrums of the gAgNPs (a), AgNPs (b), and GTE (c).

In the UV–visible spectroscopy technique, the spectrum of synthesized silver nanoparticles is recorded at the range of 400–500 nm that show a surface plasmon resonance (SPR) band with a maximum absorbance of around 420 nm, indicating the metallic silver (Shankar et al., 2021). According to the Figure 1a,b, the produced gAgNPs and AgNPs created a broad peak in the wavelength of 423 and 413 nm in the UV–visible spectrums, respectively, that can be related to the Ag synthesis, while the GTE did not show any peaks in the range 400–500 nm (Figure 1c), corresponding to the absence of silver nanoparticles in this sample. The higher broad SPR peaks in UV–Vis spectrums of the silver nanoparticles indicate their higher polydispersity index (Mohamed & Madian, 2020). According to our findings, the gAgNPs showed a higher broad SPR peak followed by a higher PDI than AgNPs. Pandey et al. (2020) reported that UV–visible spectrum of the synthesized AgNPs represented a peak at 427 nm, which is attributed to the presence of AgNPs. In another study, UV–visible spectroscopy of the prepared AgNPs showed the surface plasmon resonance peak around 400 nm corresponding to the formation of AgNPs (Selvaraj et al., 2018). Jardón‐Romero et al. (2022) observed the UV–visible spectrums of the biogenic AgNPs in the range 431–447 nm. In agreement with our results, they reported that no SPR at wavelengths above 500 nm can be related to the homogeneity and small size of the synthesized silver nanoparticles.

### 3.2 Dynamic light scattering (DLS)

**FIGURE 2:**
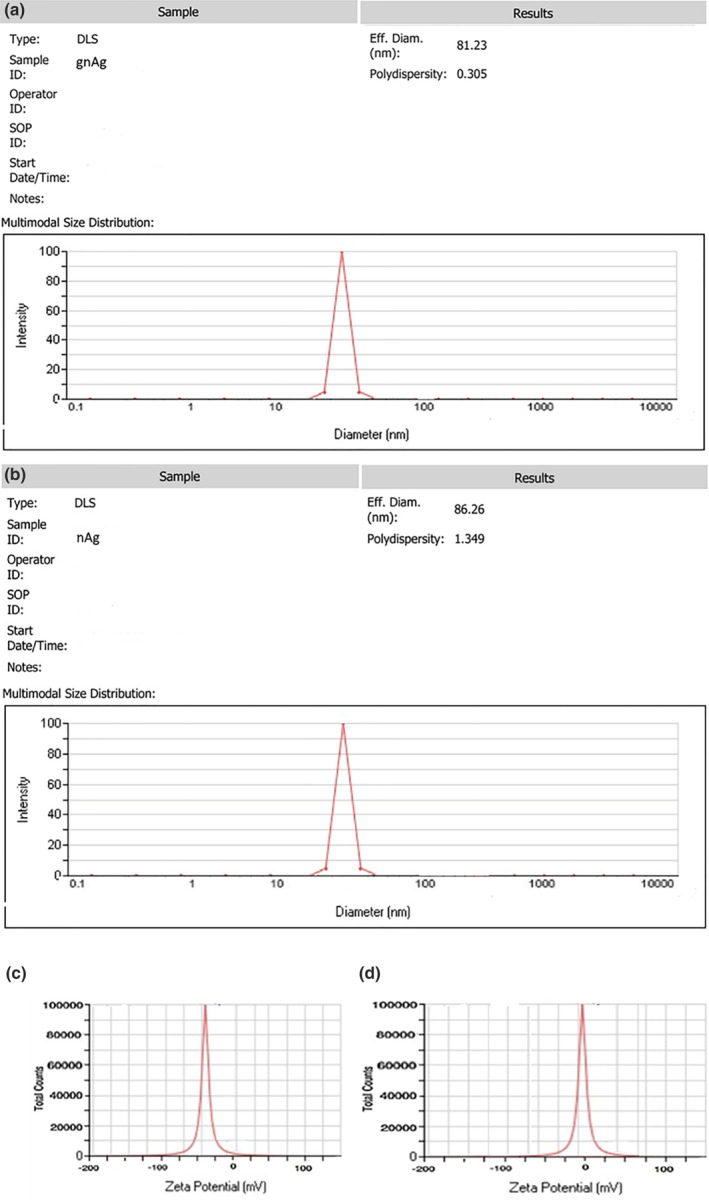
(a–d) Dynamic light scattering (DLS) findings (size, PDI, and zeta potential) of the gAgNPs (a and c) and AgNPs (b and d).

The size, polydispersity index (PDI), and zeta potential of the synthesized AgNPs and gAgNPs are represented in Figure 2a–d. According to the obtained findings, size, PDI, and zeta potential of the gAgNPs were 81.23 nm, 0.305, and −39, while those of AgNPs were 86.26 nm, 1.349, and +5, respectively. Generally, the smaller size and PDI in the range of 0.1–0.7 of the nanoparticles increase their bioactivity and efficiency. Also, zeta potential above +30 mV and below −30 mV indicates the higher stability of the produced nanoparticles. In this study, gAgNPs showed more appropriate physicochemical properties than AgNPs which can lead to the higher efficiency of the gAgNPs. It seems that the reduction of silver nitrate with GTE can produce more desirable nanoparticles than the chemical method. Kumar et al. (2021) reported that the average diameter of the fabricated AgNPs using *Plukenetia volubilis* L. seed flour was 43.4 ± 21.1 nm. They concluded that the biosynthesized gAgNPs were highly dispersed in an aqueous environment and had an average PDI of 0.2363. Hassanisaadi et al. (2022) biosynthesized silver nanoparticles with a diameter of 60.78 nm and PDI of 0.356, using the aqueous leaf extract of *Aloysia citrodora*. They reported that the biogenic AgNPs showed significant antifungal and anti‐*Pythium* activity. In another study, the average particle size of the synthesized silver nanoparticles was 100 ± 40 nm with the PDI of 0.298. They reported that this PDI shows that the synthesized silver nanoparticles have a broad distribution (Mohamed & Madian, 2020).

### 3.3 X‐ray diffraction (XRD)

**FIGURE 3:**
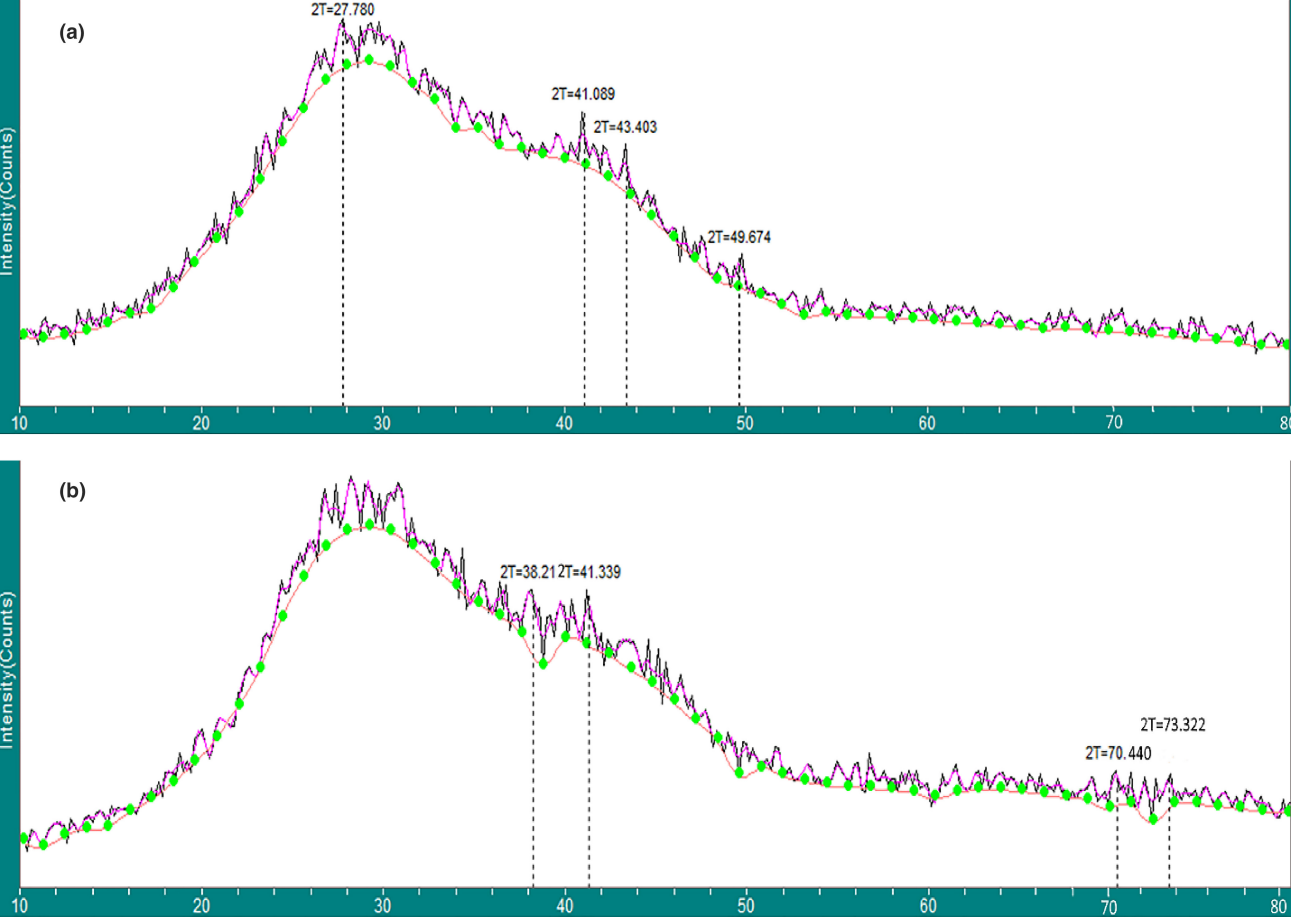
(a, b) X‐ray diffraction (XRD) graphs of the gAgNPs (a) and AgNPs (b).

The XRD spectrums of the gAgNPs and AgNPs are depicted in Figure 3a,b. According to the XRD pattern of the gAgNPs (Figure 3a), it is seen that three peaks at 2*θ* = 41.089°, 43.403° and 49.674° were obtained corresponding to the (111), (200), and (222) planes of face‐centered cubic (fcc) structures of pure silver metal. In addition, there is another peak at 2*θ* = 27.780° due to the crystallization of the phytochemical ingredients of GTE, wrapping around the gAgNPs. Kumar et al. (2021) observed two peaks at 2*θ* = 38.098° and 44.158° corresponding to the (111) and (200) planes of face‐centered cubic (fcc) structures of pure silver nanoparticles (ICSD no‐98‐005‐3761) in the obtained XRD patterns of the biosynthesized silver nanoparticles using Andean Sacha inchi seed flour (SISF). In addition, they reported that there was another broad reflection at 2*θ* = 15–32°, and undesired peaks at 27.3 and 29.7 were due to SISF's effective chemical framework, wrapping around the nanoparticles, and indicates the semi‐crystalline nature of AgNps.

The XRD pattern of the AgNPs shows the emission peaks at 2θ value at 38.219°, 41.339°, 70.44°, and 73.322°. According to the Inorganic Crystal Structure Database (ICSD, https://icsd.fi‐karlsruhe.de, No. 98‐005‐3759), the four diffraction peaks indicate the crystalline planes of (111), (200), (220), and (311), corresponding to the face‐centered cubic (fcc) structure of Ag nanoparticles, respectively (Gordon‐Falconí et al., 2020; Hassanisaadi et al., 2022). Pandey et al. (2020) reported that the crystalline nature of Ag nanoparticles appeared at 2*θ* of 38.0° and index to the (111) reflection plane (JCPDS number 04‐0783, 1991) in the XRD pattern of polyvinyl alcohol/chitosan film‐containing AgNPs.

### 3.4 Scanning electron microscope (SEM) and atomic force microscope (AFM) analyses

**FIGURE 4:**
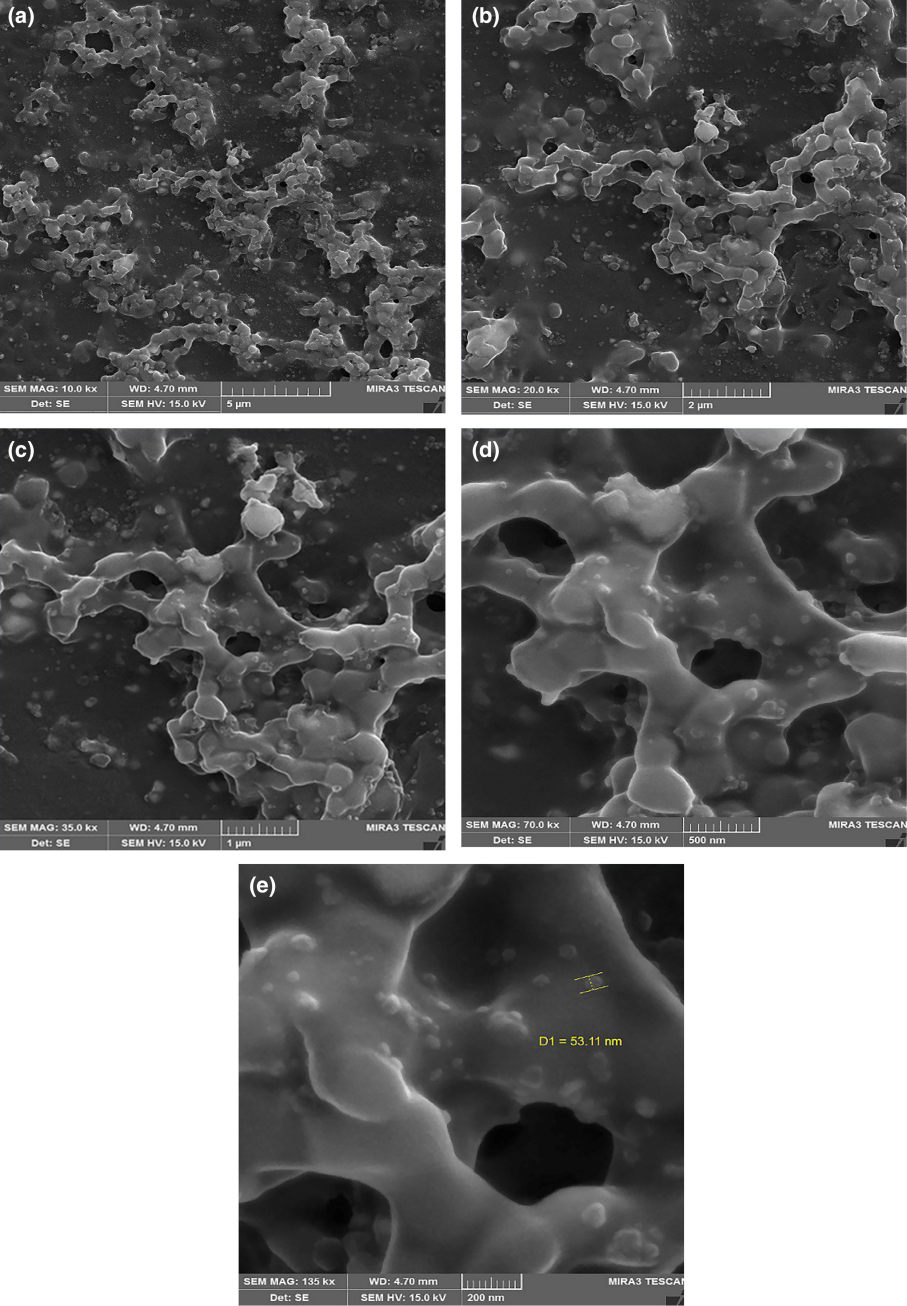
Scanning electron microscope (SEM) images of the gAgNPs in different magnifications (10, 20, 35, 70, and 135 k×).

**FIGURE 5:**
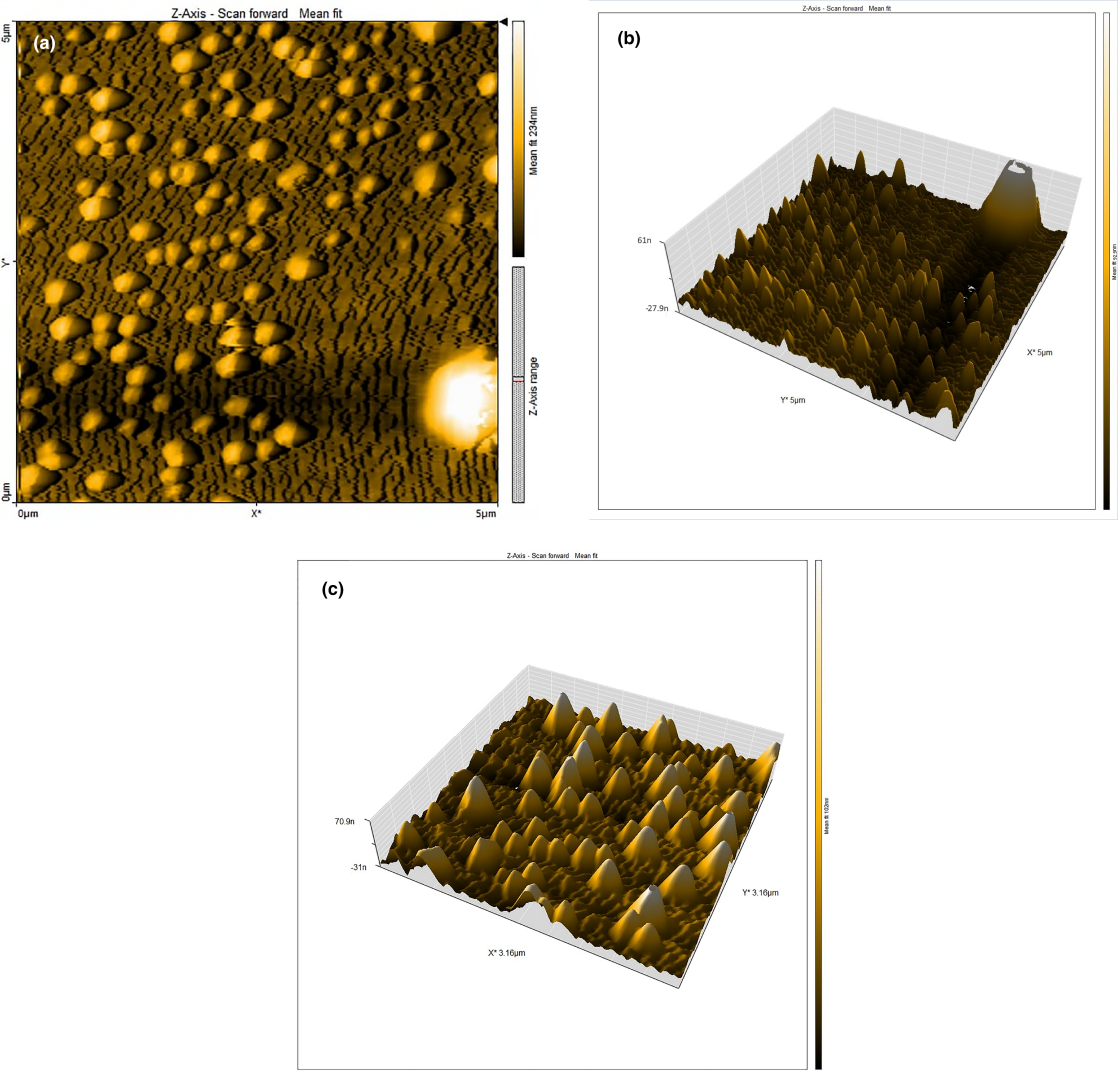
(a–c) Atomic force microscope (AFM) images of the gAgNPs (a and b) and AgNPs (c).

The SEM images of the bio‐fabricated gAgNPs are illustrated in Figure 4 in five magnifications of 10, 20, 35, 70, and 135 k×. The small spherical particles with a diameter of <50 nm can demonstrate the presence of the biofabrication of the silver nanoparticles using GTE (Hassanisaadi et al., 2022; Kumar et al., 2021). A large number of the particles are well dispersed while others are aggregated which can be related to the phytochemical matrix of GTE, which has trapped the silver particles. The AFM images of the gAgNPs and AgNPs are depicted in Figure 5a–c, respectively. Based on the SEM images, these three‐dimensional images of the produced silver nanoparticles demonstrate the spherical, uniform, and separable nanoparticles with a dimension of 53.11 nm (Abd & Hasan, 2023). According to the findings of UV–visible spectroscopy, DLS, XRD, SEM, and AFM analyses, we found that the synthesized nanoparticles were pure silver nanoparticles and crystalline in nature.

### 3.5 Microbial analysis of the wrapped turkey breast meat

#### 3.5.1 Total mesophilic bacteria

**FIGURE 6:**
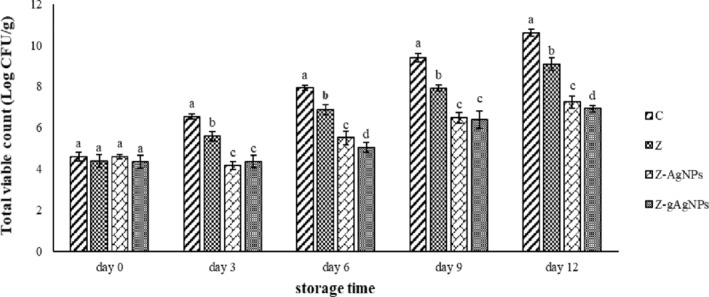
Changes in total viable count (TVC) of the packaged turkey breast meat during refrigerated storage period. Treatments: control (C) (turkey breast meat without packaging), Z (turkey breast meat packaged with zein film), Z‐AgNPs (turkey breast meat packaged with zein film containing 0.5% of AgNPs), and Z‐gAgNPs (turkey breast meat packaged with zein film containing 0.5% of gAgNPs). Different letters show a significant difference (*p* ≤ .05) among the treatments.

The total mesophilic bacteria of the wrapped turkey breast meat are illustrated in Figure 6. All of the studied treatments showed no significant difference (*p* > .05) in the bacterial population on the initial day of the storage period. By increasing the storage time, an ascending trend was observed in all of the samples so that the control group showed the highest bacterial population and Z, Z‐AgNPs, and Z‐gAgNPs were in the next ranks, respectively. Zein edible film created a thin layer around the turkey breast meat and prevented the gas and microorganism passages; as a result, microbial growth in the samples significantly (*p* ≤ .05) decreased compared to the unwrapped meat (Barkhordari & Bazargani‐Gilani, 2021; Cui et al., 2020; Sayadi et al., 2021). Also, it is clear that the package containing gAgNPs displayed the strongest antibacterial activity and could lower the bacterial count of the wrapped turkey breast meat to 6.92 log CFU/g at the end of the storage period. The value of 7.0 log CFU/g for total mesophilic bacteria is considered as the highest acceptable limit for fresh meat, to which the Z‐gAgNPs treatment did not reach during the entire storage period unlike the other treatments (Senter et al., 2000). Dissolution of the silver nanoparticles causes the release of the silver ions, which have impressive antimicrobial activity. The silver ions increase the bacterial membrane permeability leading to the disturbance in the transportation of the vital molecules in the cells, followed by the block of the respiratory enzymes, essential proteins, nucleic acid replications (DNA and RNA), and finally, cell death (Kailasa et al., 2019; Mohamed & Madian, 2020).

### 3.6 Chemical analysis of the wrapped turkey breast meat

#### 3.6.1 pH measurement

**FIGURE 7:**
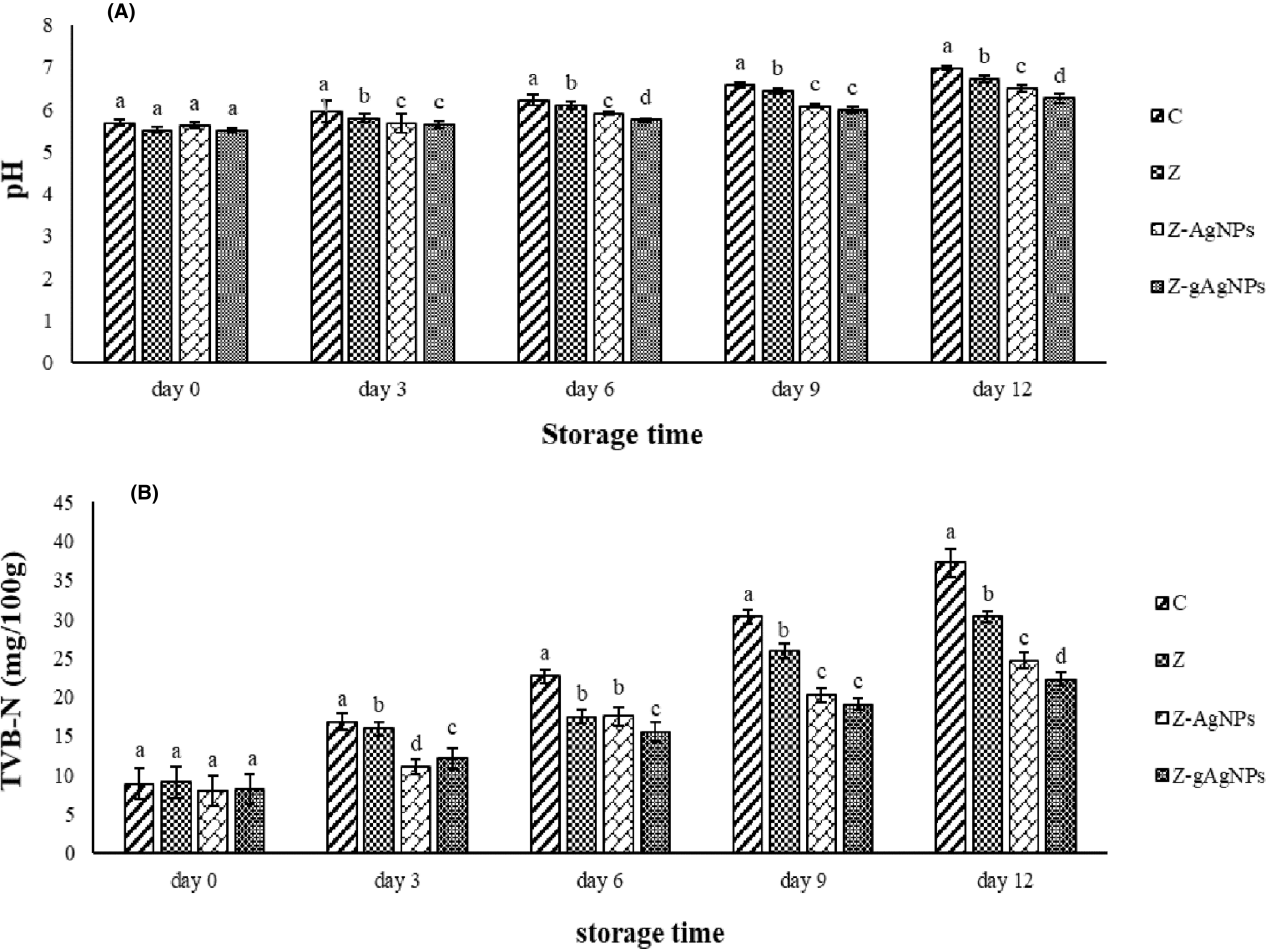
(A, B) Changes in pH (A) and total volatile basic nitrogen (TVB‐N) of the packaged turkey breast meat during refrigerated storage period. Treatments: control (C) (turkey breast meat without packaging), Z (turkey breast meat packaged with zein film), Z‐AgNPs (turkey breast meat packaged with zein film containing 0.5% of AgNPs), and Z‐gAgNPs (turkey breast meat packaged with zein film containing 0.5% of gAgNPs). Different letters show a significant difference (*p* ≤ .05) among the treatments.

pH changes in the studied samples are depicted in Figure 7A. The initial pH of all treatments was in the range 5.50–5.68 and there was no significant difference among the treatments. However, pH change trends were ascending in all of the studied treatments during storage time. The control sample showed the highest pH among the others and Z, Z‐AgNPs, and Z‐gAgNPs treatments were in the next ranks, respectively. The pH enhancement can be correlated with the enzymatic degradation of the proteins to the volatile basic compounds, such as ammonia and trimethylamine by the endogenous and exogenous (spoilage bacteria) proteases. Therefore, bacterial growth inhibition in the stored samples can decrease their pH changes during storage time (Bazargani‐Gilani et al., 2015; Haghighi & Yazdanpanah, 2020). According to Figure 7A, the packages containing AgNPs and gAgNPs could significantly (*p* ≤ .05) control pH changes of the cold‐stored turkey breast meat by their antibacterial effects so that Z‐gAgNPs treatment had the lowest pH (6.27) among the others at the end of the storage time. In one study, the inhibitory effects of the zein‐coating containing apple peel extract against pH changes in the cold‐stored chicken leg were reported. They concluded that this effect can be related to the antibacterial effects of the designated coating in the studied chicken meat during storage time (Barkhordari & Bazargani‐Gilani, 2021). Zolfaghari et al. (2023) observed the significant (*p* ≤ .05) antibacterial activity of the zein edible film containing encapsulated dill essential oil and extract in the refrigerated common carp fillets. They reported that the designated zein edible film could significantly (*p* ≤ .05) control the pH changes of the studied samples compared to the unwrapped fillets during 12 days of the storage period.

#### 3.6.2 Total volatile basic nitrogen (TVB‐N)

The TVB‐N value measures the amount of nitrogenous compounds, such as ammonia, dimethyl, and trimethylamine in meat, revealing the degree of freshness. By the spoilage of food, the protein molecules decompose to the alkaline nitrogenous compounds by the microbial and tissue proteases. As a result, the amount of this index increases. According to the standard instructions, values above 20 mg/100 g indicate food spoilage initializations (Xu et al., 2023). Figure 7B illustrates TVB‐N values of the studied treatments during the cold storage period. The initial TVB‐N value of all treatments was in the range 8.06–9.10 mg/100 g, which indicated the freshness of the used turkey breast meat. During the storage time, an ascending trend was observed in the TVB‐N values of the treatments. Then, the initial TVB‐N values of the control (8.90 mg/100 g), Z (9.10 mg/100 g), Z‐AgNPs (8.06 mg/100 g), and Z‐gAgNPs (8.22 mg/100 g) treatments increased to 37.2, 30.3, 24.7 and 22.3 mg/100 g, respectively, at the end of the storage time. An obvious significant difference (*p* ≤ .05) was observed among all treatments during the storage period. In agreement with the microbial and pH findings, the best treatment in decrease of TVB‐N value of the turkey breast was Z‐gAgNPs treatment during the storage period and Z‐AgNPs and Z were in the second and third ranks, respectively. In other words, potent antimicrobial effects of Ag nanoparticles caused the low bacterial population, followed by the low TVB‐N value in the cold‐stored turkey breast meat. Zolfaghari et al. (2023) designed an edible film based on corn zein containing encapsulated dill essential oil for the shelf‐life enhancement of the common carp fillets under refrigeration conditions. They concluded that the produced activated zein film had significant (*p* ≤ .05) antibacterial activity and therefore could prevent the increase in the TVB‐N value in the common carp fillets during storage period. Another study reported that the antibacterial coating based on the Arabic gum enriched with tomato residuum extract and dill essential oil significantly (*p* ≤ .05) decreased TVB‐N values of the rainbow trout fillets during refrigerated storage period (Tavakkoli et al., 2020).

## 4 CONCLUSION

It is concluded that the biofabricated silver nanoparticles by GTE exhibited unique characteristics, such as size, PDI, zeta potential, uniformity, and shape, compared to the chemical ones. Therefore, their bioactivity properties were more effective than the chemically synthesized silver nanoparticles. According to our findings, the package containing gAgNPs significantly (*p* ≤ .05) showed higher efficiency than AgNPs in decreasing the total viable count, pH, and TVB‐N values of the cold‐stored turkey breast meat at the end of the storage period. Considering the environmental issue and the obtained efficiency level, the usage of the biofabricated silver nanoparticles by GTE seems to be beneficial and very helpful. The comparison of the synthetic silver nanoparticles efficiency with the green synthesized ones by GTE is suggested to be considered for other applications in different fields, especially medicine.

## AUTHOR CONTRIBUTIONS

**Ammar Mohammed Ali Eesa:** Conceptualization (equal); data curation (equal); formal analysis (equal); investigation (equal); methodology (equal); software (equal); writing – original draft (equal). **behnaz Bazargani‐Gilani:** Conceptualization (equal); data curation (equal); formal analysis (equal); investigation (equal); methodology (equal); software (equal); supervision (equal); writing – original draft (equal); writing – review and editing (equal). **Shaimaa Obaid Hasson:** Conceptualization (equal); data curation (equal); formal analysis (equal); investigation (equal); methodology (equal); software (equal); supervision (equal); validation (equal).

## FUNDING INFORMATION

This research received no specific grant from any funding source.

## CONFLICT OF INTEREST STATEMENT

None.

## ETHICS STATEMENT

This study does not involve any human or animal testing.

## Data Availability

The data that support the findings of this study are available on request from the corresponding author.
